# Acute Graft-Versus-Host Disease of the Kidney in Allogeneic Rat Bone Marrow Transplantation

**DOI:** 10.1371/journal.pone.0115399

**Published:** 2014-12-26

**Authors:** Seiichiro Higo, Akira Shimizu, Yukinari Masuda, Shinya Nagasaka, Yusuke Kajimoto, Go Kanzaki, Megumi Fukui, Kiyotaka Nagahama, Akiko Mii, Tomohiro Kaneko, Shuichi Tsuruoka

**Affiliations:** 1 Department of Nephrology, Nippon Medical School, Tokyo, Japan; 2 Department of Analytic Human Pathology, Nippon Medical School, Tokyo, Japan; UNIFESP Federal University of São Paulo, Brazil

## Abstract

Allogeneic hematopoietic cell or bone marrow transplantation (BMT) causes graft-versus-host-disease (GVHD). However, the involvement of the kidney in acute GVHD is not well-understood. Acute GVHD was induced in Lewis rats (RT1^l^) by transplantation of Dark Agouti (DA) rat (RT1^a^) bone marrow cells (6.0×10^7^ cells) without immunosuppression after lethal irradiation (10 Gy). We examined the impact of acute GVHD on the kidney in allogeneic BMT rats and compared them with those in Lewis-to-Lewis syngeneic BMT control and non-BMT control rats. In syngeneic BMT and non-BMT control rats, acute GVHD did not develop by day 28. In allogeneic BMT rats, severe acute GVHD developed at 21–28 days after BMT in the skin, intestine, and liver with decreased body weight (>20%), skin rush, diarrhea, and liver dysfunction. In the kidney, infiltration of donor-type leukocytes was by day 28. Mild inflammation characterized by infiltration of CD3+ T-cells, including CD8+ T-cells and CD4+ T-cells, and CD68+ macrophages to the interstitium around the small arteries was noted. During moderate to severe inflammation, these infiltrating cells expanded into the peritubular interstitium with peritubular capillaritis, tubulitis, acute glomerulitis, and endarteritis. Renal dysfunction also developed, and the serum blood urea nitrogen (33.9±4.7 mg/dL) and urinary N-acetyl-β-D-glucosaminidase (NAG: 31.5±15.5 U/L) levels increased. No immunoglobulin and complement deposition was detected in the kidney. In conclusion, the kidney was a primary target organ of acute GVHD after BMT. Acute GVHD of the kidney was characterized by increased levels of urinary NAG and cell-mediated injury to the renal microvasculature and renal tubules.

## Introduction

Allogeneic hematopoietic cell transplantation (HCT) is a clinical treatment for a variety of conditions, including hematologic disorders, metabolic storage diseases, immune deficiencies, and is used as a rescue technique after cancer treatment [1], [2]. Despite improved outcomes following HCT, renal impairments remain a common complication. Acute kidney injury has been reported to manifest in approximately 70% of HCT recipients [3]. Acute kidney injury itself is an important risk factor for the development of chronic kidney disease, and is associated with increased short- and long-term mortality following HCT [4]. Therefore, strategies to preserve renal function in patients receiving HCT should be implemented, given the potential for positive patient outcomes. Often, the accurate etiology of post-transplant renal dysfunction cannot be diagnosed, as renal biopsy is rarely performed in the peri-transplantation period.

In patients with HCT, multiple factors have been linked to the development of renal impairments, including preexisting renal injury, direct effects of conditioning chemotherapy and irradiation, complications of the infused cryopreserved cells, tumor lysis syndrome, calcineurin inhibitor used for graft-versus-host disease (GVHD) prophylaxis, and infection and its treatment [3], [5]–[7]. Despite the multiple etiologies of post-transplant renal dysfunction, GVHD has rarely been linked to the kidney, and most physicians believe that the kidney is not a target of acute GVHD. However, several recent studies have demonstrated chronic GVHD of the kidney that resulted in nephrotic syndrome [8], [9]. In addition, some studies suggest that acute GVHD may also develop in the kidney after HCT [10], [11].

In the present study, to clarify whether acute GVHD develops in the kidney, we used the major histocompatibility complex–disparate rat allogeneic bone marrow transplantation (BMT) model. We used the already established rat GVHD model, which involves transplantation of bone marrow cells (BMCs) from DA rats (RT1^a^, RT1A^a^) into lethally irradiated Lewis rat (RT1^l^, RT1A^l^) recipients without immunosuppression [12]. Although, this rat BMT model is different from clinical HCT in human, this model is considered to be useful to evaluate the acute GVHD on the kidney, because severe and acute GVHD develops within 21 days after BMT in this model.

## Materials and Methods

### Animals

The animal experiments described in this study were approved by the Animal Experiments Ethical Review Committee of Nippon Medical School (protocol no. 26–122). We used inbred male DA and Lewis rats (Charles River Japan, Kanagawa, Japan) that weighed 190–220 g and 220–270 g, respectively. All animals received humane care in compliance with the Guideline by the Committee of Nippon Medical School.

### Bone Marrow Transplantation

BMC suspensions were harvested from DA and Lewis rats by flushing the marrow from the femurs and tibias with cold RPMI 1640 (Life Technologies, Grand Island, NY) supplemented with 2.5% fetal bovine serum and 25 mM HEPES. Recipient Lewis rats were irradiated with a dose of 10 Gy (MBR 1505R2; type PI-CR-1505R2, type MI-RC-3E, Hitachi Medical Corporation, Japan) prior to BMT. After 2–3 h, 6.0×10^7^ BMCs from the DA or Lewis rats were then injected into Lewis rat recipients via the tail vein.

In this model, acute GVHD developed by day 21 to day 28 in allogeneic BMT rats. The growth of transplanted BMCs, body weight, degree of acute GVHD, liver and renal functions, pathology, and cytokines milieu were evaluated by day 28 in allogeneic BMT rats (n = 5 at each time point), Lewis-to-Lewis syngeneic BMT control rats (n = 5 at each time point), and non-BMT control rats (n = 3 at each time point).

### Reconstruction of Transplanted BMCs

To examine the reconstruction of transplanted BMCs, blood samples were collected on days 4, 7, 14, 21, and 28 after BMT from the tail vein, to measure the number of white blood cells (Full Automatic Blood Cell Counter, model: PCE-210N, ERMA. INC), and flow cytometry was conducted to assess the expression of RT1A^a^ (donor-type cells), CD6+ T-cells, CD8+ T-cells, CD4+ T-cells, and CD68+ macrophages. Peripheral blood mononuclear cells were treated with anti-mouse CD16/32 Ab (clone 2.4G2) to block the Fc-receptors followed by direct or indirect staining of fluorochrome-conjugated antibodies (Table 1). Dead cells were identified and excluded using propidium iodide. Cell suspensions were analyzed on a FACSCanto II flow cytometer (BD Biosciences).

**Table 1:** The kinds of reagents.

*	Reagent	Target	Clone	Conjugate	Company
Flow, IF	Anti-rat RT1A^a,b^ Ab	MHC class I	C3	FITC	BioLegend, San Diego, CA
IF	Anti-rat CD3 Ab	T cell	1F4	FITC	BioLegend, San Diego, CA
IF	Anti-rat CD4 Ab	CD4+ T cell	10B5	Purified	Abcam, Tokyo, Japan
Flow	Anti-rat CD4 Ab	CD4+ T cell	W3/25	APC-Cy7	BioLegend, San Diego, CA
Flow	Anti-rat CD6 Ab	T cell	OX-52	FITC	BD Pharmingen, San Diego, CA
Flow, IF	Anti-rat CD8α Ab	CD8+ T cell	G28	PE	BioLegend, San Diego, CA
IF	Anti-rat CD45 Ab	White blood cell	OX-1	PE-Cy7	BioLegend, San Diego, CA
IHC	Anti-human CD3 Ab	T cell	Rabbit, Polyclonal	Purified	DAKO, Glostrup, Denmark
IHC	Anti-rat CD8α Ab	CD8+ T cell	OX-8	Purified	BioLegend, San Diego, CA
IHC	Anti-rat CD68 Ab	Macrophage	ED1	Purified	BMA BIOMEDICALS, Augst, Switzerland
Flow	Anti-rat Granulocyte Marker Ab	Monocyte, Macrophage	HIS48	Biotin	eBioscience, San Diego, CA
Flow	Streptavidin			PE-Cy7	BioLegend, San Diego, CA
Flow, IF	Anti-mouse CD16/32 Ab	Fcγ receptor III/II	2.4G2	Culture sup	ATCC**, Manassas, VA
IF	Anti-mouse IgG1 Ab	IgG1	Goat, Polyclonal	TRITC***	SouthernBiotech, Birmingham, AL
IF	Anti-rat IgM Ab	IgM	Goat, Polyclonal	FITC	MP Biomedicals, Tokyo, Japan
IF	Anti-rat IgG Ab	IgG	Rabbit, Polyclonal	FITC	MBL, Nagoya, Japan
IF	Anti-rat C3 Ab	C3	Goat, Polyclonal	FITC	MP Biomedicals, Tokyo, Japan
IF	Anti-rat RT1B Ab	MHC class II	OX-6	PE	BioLegend, San Diego, CA

*Assessment: Flow: Flow cytometory; IHC: Immunohistochemistry; IF: Immunofluorescence.

**American Type Culture Collection.

***Tetramethylrhodamine isothiocyanate.

### Systemic Analysis of GVHD

The degree of systemic GVHD was assessed using a standard scoring system that incorporated five clinical parameters: weight loss, posture (hunching), activity, fur texture, and skin integrity [13]. Each parameter was evaluated and graded from 0 to 2. A clinical index was subsequently generated by the sum of the five criteria scores (maximum index  = 10). The skin, liver, intestine, and kidney from allogeneic BMT rats were examined pathologically at day 28 after BMT. As controls, the skin, liver, intestine, and kidney from non-BMT control Lewis rats and from Lewis-to-Lewis syngeneic BMT control rats were prepared at day 28 after BMT. Blood samples were collected on day 28 from allogeneic and syngeneic BMT rats and non-BMT control rats to examine liver function (total bilirubin [T-Bil], aspartate aminotransferase [AST], and alanine aminotransferase [ALT]), as well as renal function (serum creatinine [Cr], blood urea nitrogen [BUN]), using an autoanalyzer (SRL, Tokyo, Japan). Urine was also collected on day 28, to examine proteinuria and urinary N-acetyl-β-D-glucosaminidase (NAG) levels (SRL, Tokyo, Japan).

### Pathology and Immunohistochemistry

To study the histological features of GVHD, the skin, liver, intestine, and kidney tissues were fixed in 20% buffered formalin and embedded in paraffin for light microscopic examination. Tissues were stained with hematoxylin and eosin (H&E) and periodic and acid-Schiff (PAS) staining for histopathological examination, and naphthol AS-D chloroacetate esterase staining to detect neutrophils. The primary antibodies used for immunohistochemistry are indicated in Table 1. To detect infiltrating CD3+ T-cells, CD8+ T-cells and CD68+ macrophages, 20% -buffered, formalin-fixed, paraffin-embedded tissue sections were stained by the standard avidin-biotin-peroxidase complex technique. Before incubation with the primary antibody, for ED1+ and CD3+ detection, tissue sections were incubated with 0.1% pepsin for 45 min. For CD8+ detection, the sections were treated with 1 mM EDTA (pH 8.0) in hot water at 90°C for 3 h.

In each kidney sample, the number of infiltrating CD3+ T-cells, CD8+ T-cells, and CD68+ macrophages was examined in 40 randomly selected interstitial fields at ×200 magnification, by an investigator blinded to the clinical or histological findings.

Immunofluorescence study was also performed using frozen tissues. The deposition of IgM, IgG, and C3 was evaluated using the indirect method. To detect the donor type of leukocytes in the kidney, double stain with fluorescein isothiocyanate (FITC)-conjugated anti-rat RT1A^a,b^ antibody (donor type of RT1A^a^) and phycoerythrin (PE)-conjugated anti-rat CD45 antibody (leukocyte common antigen) was performed. To detect CD8+ T-cells or CD4+ T-cells, double stain with FITC-conjugated anti-rat CD3 antibody and PE-conjugated anti-rat CD8α antibody or anti-rat CD4 antibody was performed. To evaluate the expression of MHC class II in renal tubules, immunostaining with PE-conjugated anti-rat RT1B antibody was performed. In each kidney sample, more than 100 cross-sections of renal tubules were graded semiquantitatively based on the specimens stained for rat RT1B, using the following grading system: absence of MHC class II staining; 0, mild increase of MHC class II staining; 1, moderate increase of MHC class II staining; 2, marked increase of MHC class II staining; 3.

### Real-time Reverse Transcription-Polymerase Chain Reaction for Cytokines

Real-time reverse transcription-polymerase chain reaction (Real-time RT-PCR) was performed as described previously [14], to examine the mRNA expression levels of interferon (IFN)-γ, tumor necrosis factor (TNF)-α, interleukin (IL)-4, and IL-17 in the kidney. Total renal RNA was extracted using the ISOGEN (Nippon Gene, Japan) according to the manufacturer's protocol. The purified total RNA was 1.9–2.2 of A260/A280. cDNA libraries were created with a High Capacity cDNA Reverse Transcription kit (Applied Biosystems, Foster City, CA) according to manufacturer's protocol from 2 µg of total RNA. The gene expression levels were analyzed by quantitative RT-PCR using the THUNDERBIRD SYBR qPCR Mix (TOYOBO, Osaka, Japan) according to the manual supplied by the manufacturer (ABI PRISM 7900 HT, Applied Biosystems). The normalized value for mRNA expression in each sample was calculated as the relative quantity of relevant primers divided by the relative quantity of the housekeeping gene, β-actin. The RT-PCR primer sequences used in this study are listed in Table 2. Quantification was performed using the SDS 2.3 software program (Applied Biosystems).

**Table 2:** Primer sequences used for the gene expression analysis.

Gene	Forward primer sequence (5-3′)	Reverse primer sequence (5-3′)
IFN-γ	GAACTGGCAAAAGGACGGTA	CTGATGGCCTGGTTGTCTTT
TNF-α	AAATGGGCTCCCTCTCATCAGTTC	TCTGCTTGGTGGTTTGCTACGAC
IL-4	TCCTTACGGCAACAAGGAAC	GTGAGTTCAGACCGCTGACA
IL-17	ATCAGGACGCGCAAACATG	TGATCGCTGCTGCCTTCAC
β-actin	ACCACCATGTACCCAGGCATT	CCACACAGAGTACTTGCGCTCA

### Statistical Analysis

The results were expressed as mean ± standard deviation. Differences were evaluated using either the Student *t*-test using an analytical software program (Excel, Microsoft, Redmond, WA) or ANOVA determined by analysis of variance which performed Fisher's one. Differences were considered significant at the 95% confidence intervals (P<0.05).

## Results

### Characterization of Circulating Leukocytes in Peripheral Blood

**Figure 1:**
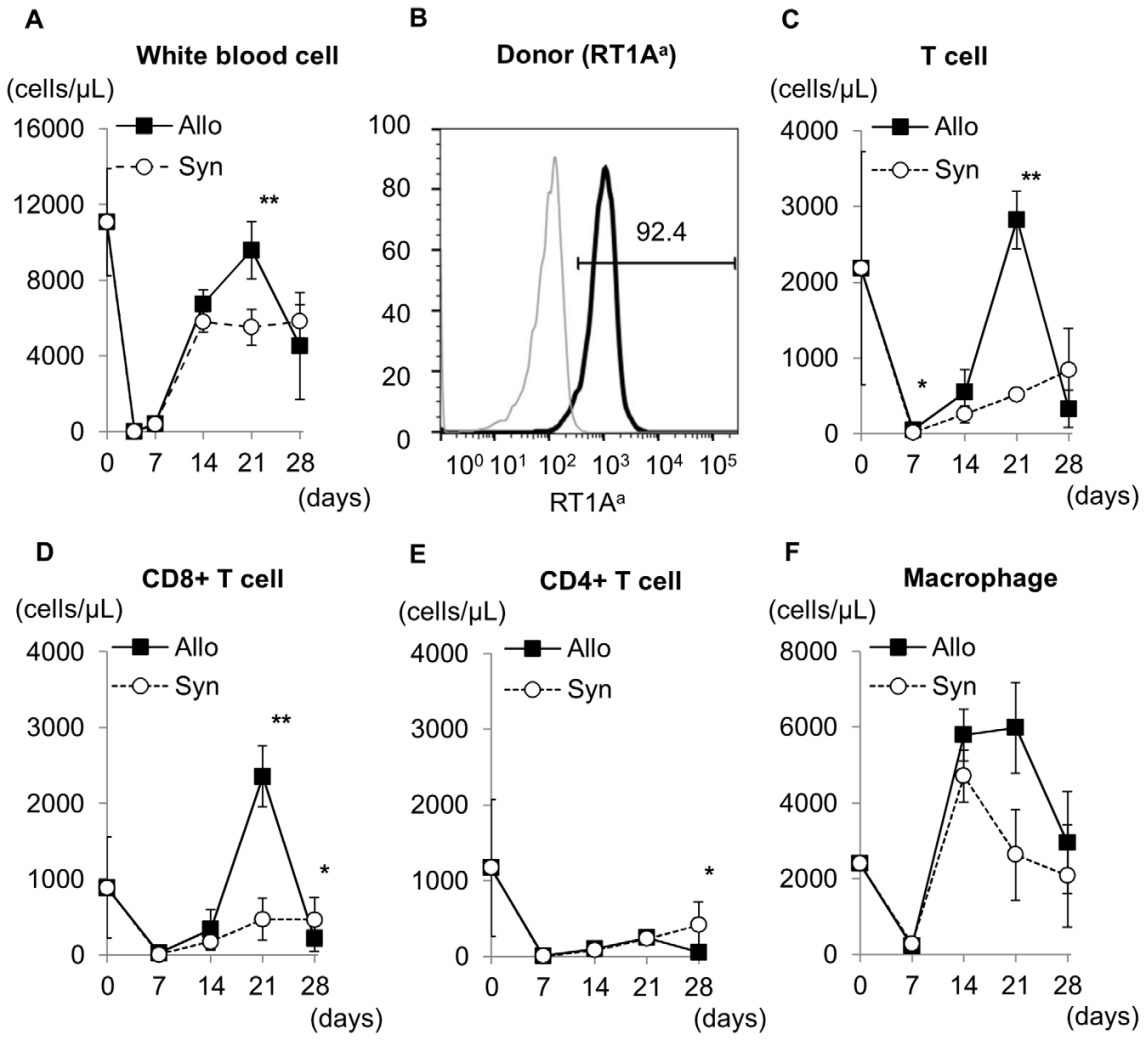
Leukocytes in the peripheral blood after bone marrow transplantation (BMT). The total white blood cell (WBC) count in peripheral blood (A) decreased markedly on day 4, but recovered between day 7 and day 14 in both allogeneic and syngeneic BMT rats. The number of WBCs in the peripheral blood was higher on day 21 in allogeneic BMT rats than in syngeneic BMT rats. WBCs in the peripheral blood decreased again in allogeneic BMT rats on day 28, which may be because of recruitment of WBCs to GVHD organs. Almost all circulating leukocytes in allogeneic BMT rats on day 28 after BMT (B) expressed donor-type RT1A^a^, indicating that circulating leukocytes in peripheral blood originated from the donor (Gray; no staining, Black; anti-RT1A^a,b^). In peripheral blood, CD6+ T-cells (C), CD8+ T-cells (D), CD4+ T-cells (E), and ED1+ macrophages levels (F) recovered between day 7 and day 21 after BMT in both syngeneic and allogeneic BMT rats. The number of CD6+ T-cells and CD8+ T-cells was significantly higher on day 21 in allogeneic BMT rats than in syngeneic BMT rats. The number of CD4+ T-cells and CD68+ macrophages was similar in both syngeneic and allogeneic BMT rats. *P<0.05, **P<0.01.

After 10 Gy irradiation and allogeneic or syngeneic BMT, circulating leukocytes markedly decreased (by>95%) on day 7, but recovered by day 14 (Fig. 1A). In allogeneic BMT rats, circulating leukocyte levels approached control levels on day 21, however, then decreased by day 28. By contrast, circulating leukocytes in syngeneic BMT rats gradually recovered by day 28. Almost all circulating mononuclear cells in peripheral blood in allogeneic BMT rats originated from the donor transplant (Fig. 1B).

In syngeneic BMT rats, circulating CD6+ and CD8+ T-cell levels gradually recovered by day 28 (Fig. 1C and 1D). In allogeneic BMT rats, CD6+ and CD8+ T-cell levels recovered from day 14, and the number of CD6+ and CD8+ T-cells was significantly higher on day 21 than in syngeneic BMT rats. Thereafter, CD6+ and CD8+ T-cell levels decreased by day 28, which might be associated with recruitment to the GVHD organs. The number of CD4+ T-cells and CD68+ macrophages was similar in the peripheral blood of allogeneic and syngeneic BMT rats (Fig. 1E and 1F).

### Development of Systemic Acute GVHD

**Figure 2:**
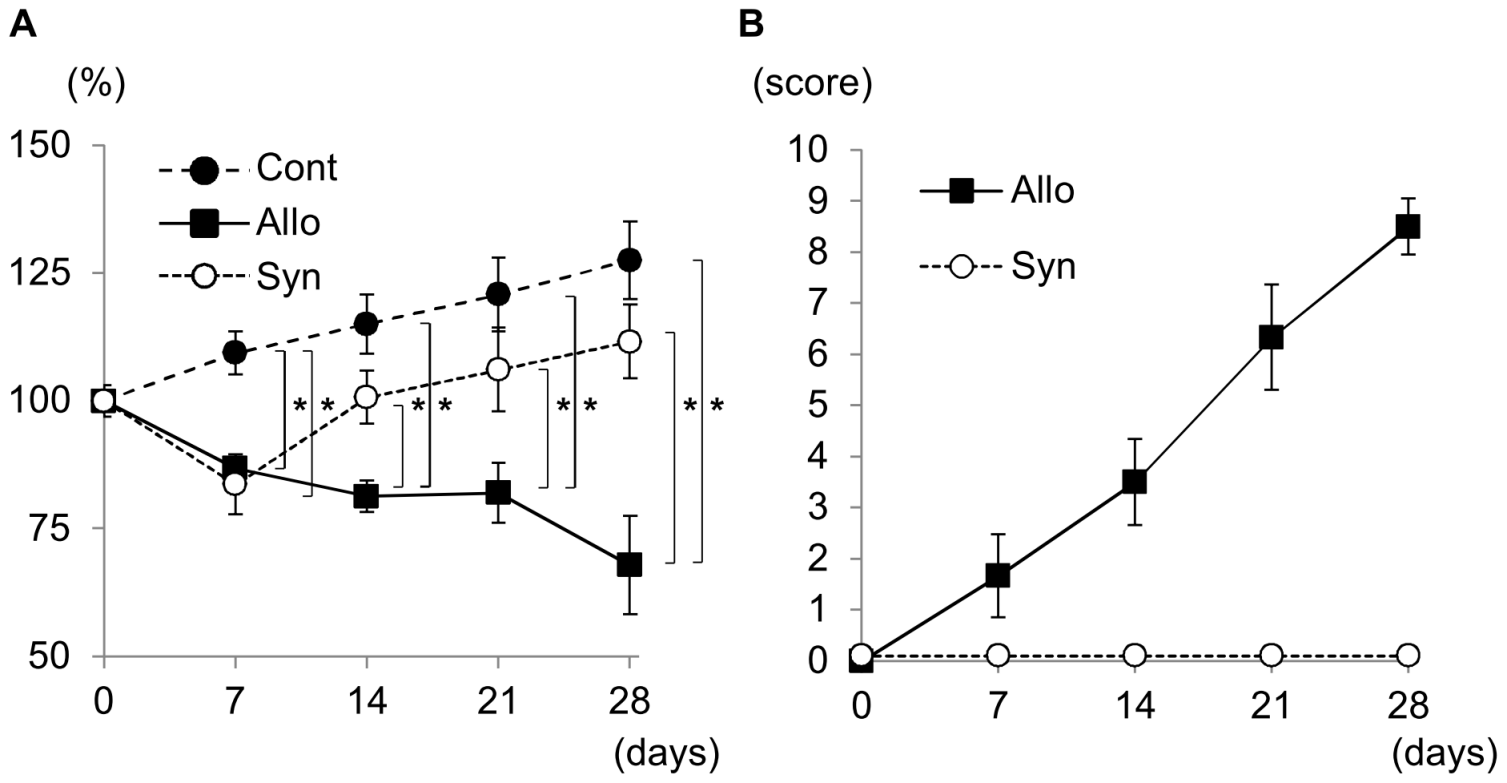
Body weight and semiquantitative score of systemic acute GVHD after bone marrow transplantation (BMT). Comparison of body weight in percentage (A) on day 0, after radiotherapy and after BMT showed that this parameter decreased in syngeneic and allogeneic BMT rats on day 7 and continued gradually to decrease in allogeneic BMT rats by>20% on day 28. In addition, body weight was significantly lower in allogeneic BMT rats than in syngeneic BMT rats between day 14 and day 28 after BMT. The semiquantitative score of systemic acute GVHD (B) showed that symptoms associated with acute GVHD occurred from day 7 in allogeneic BMT rats, and developed by day 28 with score of 8.7±0.5 (mean ± SD; score 0–10). In contrast, acute GVHD did not develop in syngeneic BMT rats by day 28. *P<0.05.

**Figure 3:**
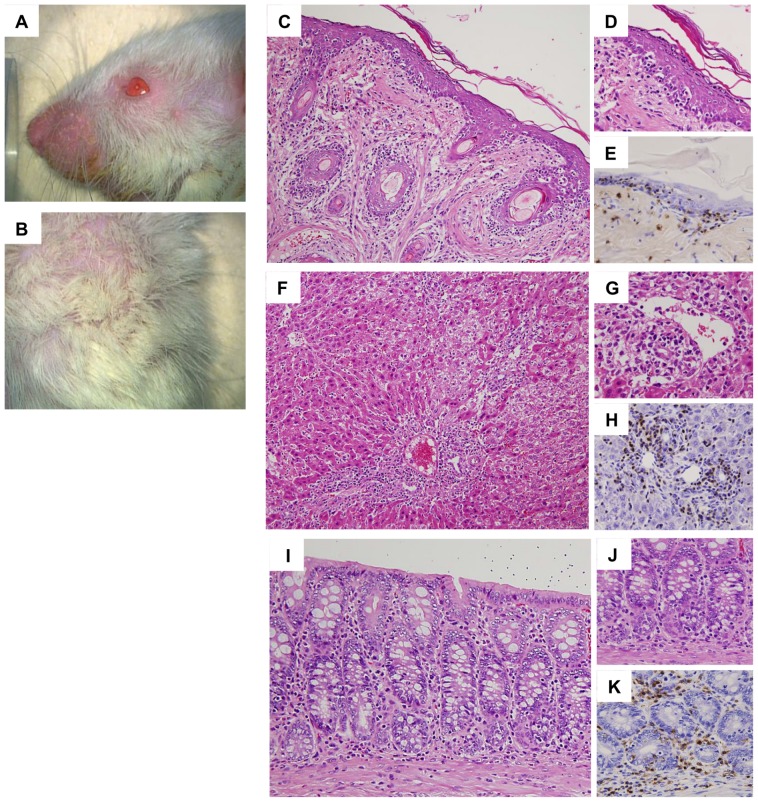
Acute GVHD in the skin, liver, and intestine after allogeneic bone marrow transplantation (BMT). On day 28 after allogeneic BMT, macroscopic findings of the skin indicated erythematous rush and alopecia (A, B). Light microscopic findings showed inflammatory cells, mainly CD3+ T-cells, infiltrating the epidermis and the hair follicle in the dermis, indicating acute GVHD in the skin (C, D: HE stain, E: CD3 stain, C: ×400, D, E: ×800). Inflammatory cells, mainly CD3+ T-cells, infiltrated the portal areas and spread to the hepatic lobules with cholangiolitis and phlebitis in the portal and central veins, indicating acute GVHD in the liver (F, G: HE stain, H: CD3 stain, F: ×400, G, H: ×800). In the colon, erosion and inflammatory cell infiltration was noted with cryptitis and infiltration of CD3+ T-cells, indicating acute GVHD in the colon (I, J: HE stain, K: CD3 stain, I: ×600, J, K: ×800).

**Figure 4:**
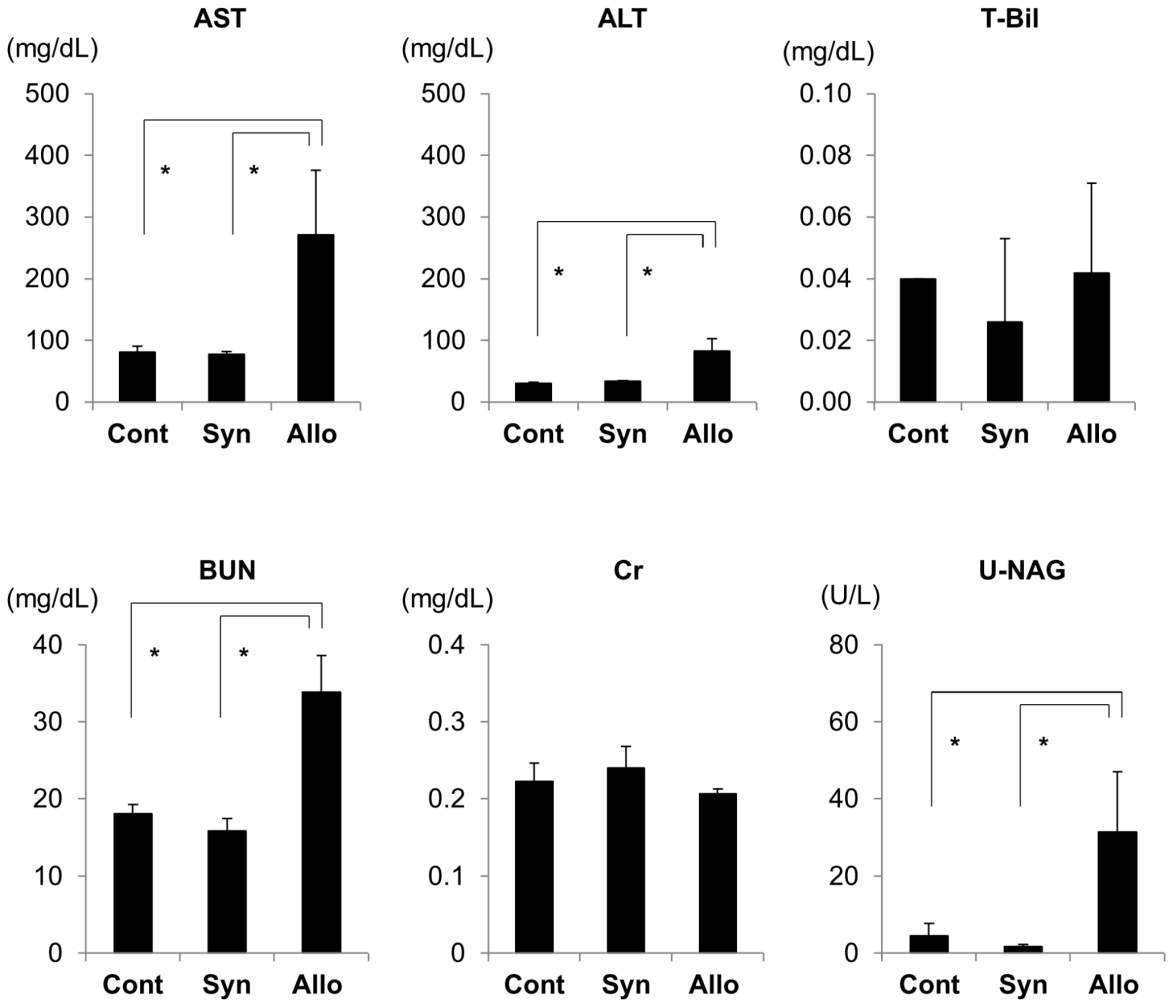
Liver and renal dysfunction after allogeneic bone marrow transplantation (BMT). Liver function was determined using serum aspartate aminotransferase (AST), alanine aminotransferase (ALT), and total bilirubin (T-Bil) levels. Serum AST and ALT levels on day 28 were significantly increased in allogeneic BMT rats compared with those in syngeneic BMT rats, although serum T-Bil levels were similar in both groups. Renal function was assessed using serum blood urea nitrogen (BUN), serum creatinine (Cr), and urinary N-acetyl-β-D-glucosaminidase (NAG) levels. Serum BUN and urinary NAG levels on day 28 significantly increased in allogeneic BMT rats compared with those in syngeneic BMT and non-BMT control rats, although serum Cr levels were similar in these groups. Importantly, urinary NAG levels in allogeneic BMT rats were increased while the serum Cr levels were stable. *P<0.05.

After DA to Lewis allogeneic BMT, the body weight of Lewis recipient rats gradually decreased by day 28 by>20% compared with that of pretransplantation (Fig. 2A). By contrast, in non-BMT control rats and Lewis-to-Lewis syngeneic BMT control rats, the body weight gradually increased by day 28, although body weight decreased transiently in syngeneic BMT rats similar to that in allogeneic BMT rats on day 7. Based on macroscopic evaluations of allogeneic BMT rats, dermatitis occurred around day 21 and developed on day 28 with erythematous rush and alopecia (Fig. 3A and 3B). Diarrhea was also noted starting from day 21 to day 28. Liver function abnormalities were also detected with increased serum AST and ALT levels (Fig. 4), although T-Bil levels were within the normal range. The semiquantitative score of systemic acute GVHD gradually increased by day 28 (Fig. 2B). Light microscopic findings of the skin, liver, and intestine on day 28 demonstrated severe acute GVHD with infiltration of CD3+ T-cells (Fig. 3C-K). Only a minimal esterase+ neutrophils were present in inflammation (data not shown). Based on these clinical signs, laboratory data, and pathology, severe acute GVHD developed in the skin, liver, and digestive tract by day 28 after BMT in the DA-to-Lewis allogeneic BMT model. However, in the Lewis-to-Lewis syngeneic BMT rats and non-BMT control rats, only few CD3+ T-cells infiltrated the skin, liver, and digestive tract, and acute GVHD did not develop by day 28 (data not shown).

### Development of Acute GVHD of the Kidney

In conjunction with the development of acute GVHD in the skin, liver, and digestive duct, renal function abnormalities developed by day 28. Serum BUN and urinary NAG levels increased on day 28 (Fig. 4), indicating renal dysfunction and proximal renal tubular injury. Urinary NAG levels were significantly increased in allogeneic BMT rats on day 28 when serum creatinine (Cr) levels were within normal range. These findings indicated that the increase in urinary NAG levels was an early and sensitive marker of acute GVHD of the kidney that occurred before the increase in serum Cr levels. Urinary protein levels were not significantly different between non-BMT control rats (709.7±72.3 mg/day), syngeneic BMT control rats (809.5±174.8 mg/day), and allogeneic BMT rats (604.6±141.4 mg/day).

**Figure 5:**
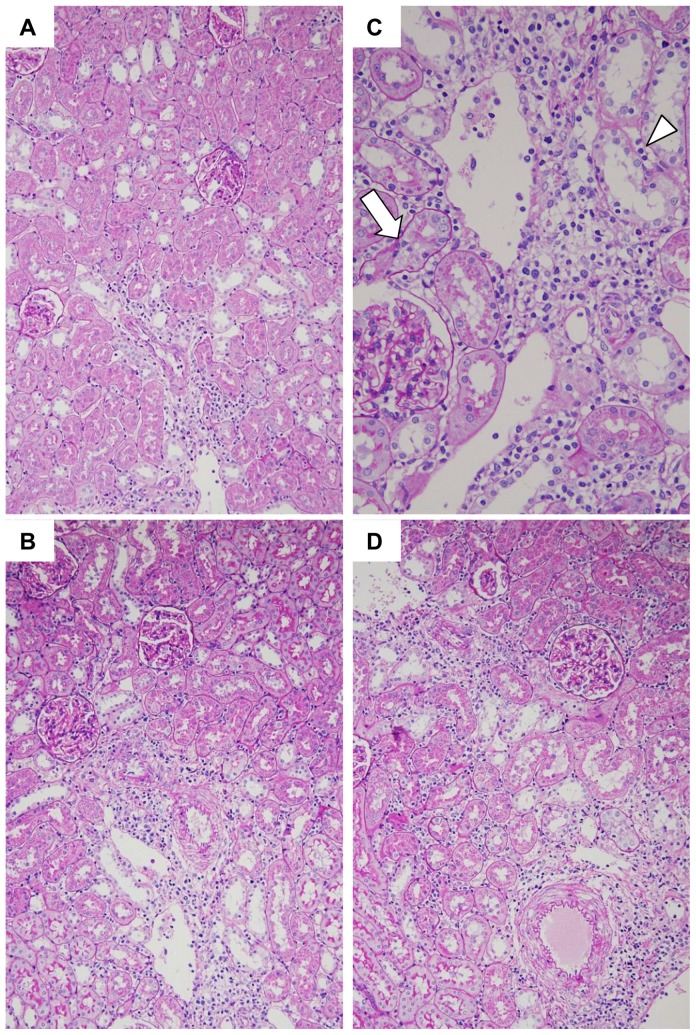
Renal inflammation in acute GVHD after allogeneic bone marrow transplantation (BMT). In mild renal inflammation (A, PAS stain, ×200), mononuclear cells infiltrated the renal interstitium around the small vessels (arrow). In moderate renal inflammation (B, PAS stain, ×200), infiltration of mononuclear cells expanded into the peritubular interstitium. High magnification of renal inflammation (C, PAS stain, ×400), interstitial cell inflammation was often accompanied by tubulitis (arrow) and peritubular capillaritis (arrowhead). In severe renal inflammation (D, PAS stain, ×200), diffuse interstitial inflammation was noted in the renal cortex.

**Figure 6:**
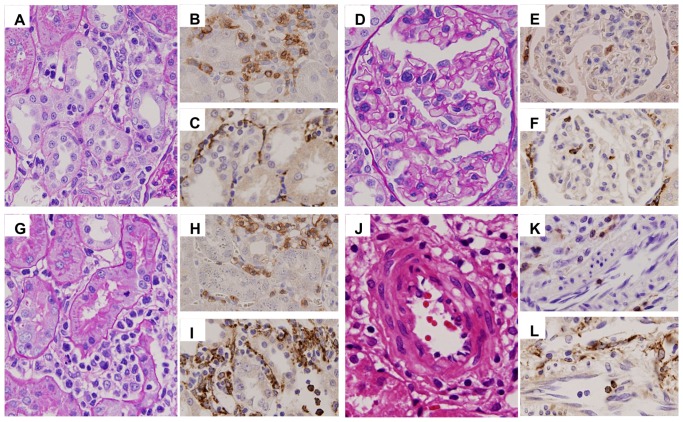
Histopathological features in the kidney on day 28 after allogeneic bone marrow transplantation (BMT). In the kidney, 28 days after allogeneic BMT, tubulitis (A–C), peritubular capillaritis (D–F), acute glomerulitis (G–I), and endarteritis (J–L) were caused by the infiltration of mononuclear cells (A, D, G, J), CD3+ T-cells (B, E, H, K), and ED-1+ macrophages (C, F, I, L), indicating cell-mediated renal injury in acute GVHD. (A, D, G: PAS stain, ×600; J: HE stain, ×1000; B, E, H, K: CD3 stain, ×600; C, F, I, L: ED1 stain, ×600).

Pathology of the kidney with acute GVHD indicated mononuclear cell infiltration to the interstitium (Fig. 5). Acute GVHD with mild renal inflammation was characterized by infiltration of mononuclear cells to the interstitium, mainly around small arteries and veins. Acute GVHD with moderate to severe renal inflammation was characterized by infiltration of inflammatory cells, which gradually expanded from the interstitium around small arteries to the peritubular interstitium. During mild to moderate renal inflammation, peritubular capillaritis and tubulitis was noted with infiltration of CD3+ T-cells and CD68+ macrophages (Fig. 5C). In addition, acute glomerulitis and acute endarteritis also developed in the kidney with marked renal inflammation (Fig. 6).

**Figure 7:**
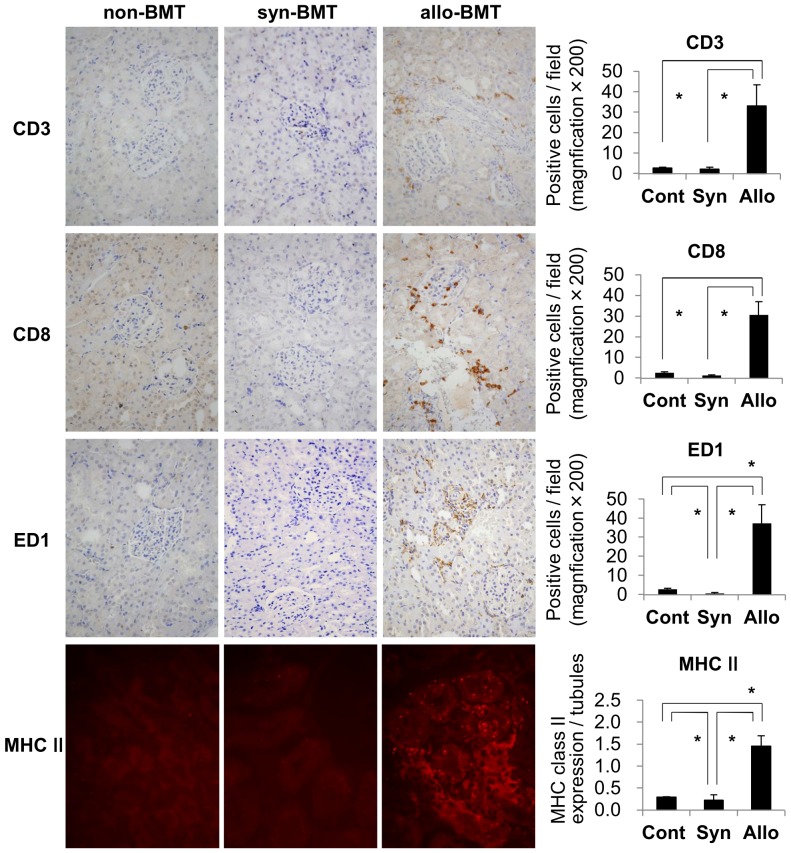
The infiltrating cells in the kidney and the MHC class II expressions in renal tubules. In the kidney on day 28 in allogeneic (allo-BMT) bone marrow transplantation rats, CD3+ T-cells including CD8+ T-cells, and ED1+ macrophages infiltrated the interstitium. The number of CD3+ T-cells, CD8+ T-cells, and macrophages per ×200 magnification field on day 28 showed that infiltration of these cells in the kidney significantly increased in allogeneic BMT rats compared with that in the non-transplanted (non-BMT) control rats and syngeneic (syn-BMT) bone marrow transplantation control rats. In addition, the expression of MHC class II in renal tubules increased in the kidney on day 28 in allogeneic BMT rats. The expression of MHC class II in renal tubules was significantly increased in allogeneic BMT rats than those in non-BMT control and syngeneic BMT control rats. *P<0.05.

**Figure 8:**
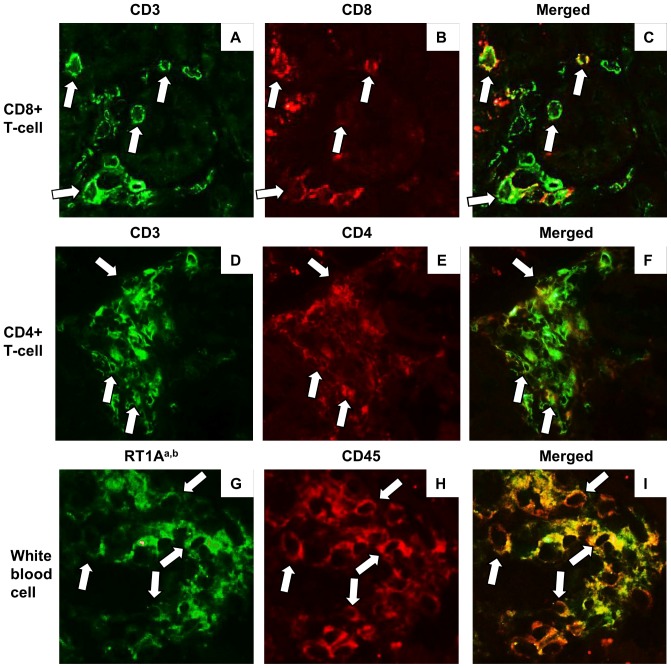
Infiltrating cells in the kidney in acute GVHD after allogeneic bone marrow transplantation (BMT). Double immunofluorescence stain by fluorescence antibody technique against CD3+ (A) and CD8+ (B), and their merged image (C) indicated that, in the kidney with acute GVHD on day 28, CD8+ T-cells (arrow in A–C) infiltrated the kidney. In addition, CD4+ T-cells (arrow in D–F) were also noted in inflammation, indicating that not only class I-restricted T cell-mediated reactions but also class II-restricted T cell-mediated reactions developed in renal acute GVHD. Double immunofluorescence stain against RT1A^a,b^ (G) and CD45 (H), and their merged image (I) indicated that, in the kidney with acute GVHD on day 28, almost all CD45+ leukocytes (arrow in G–I) were expressed rat RT1A^a, b^, suggesting the infiltration of donor-type leukocytes in acute renal GVHD.

**Figure 9:**
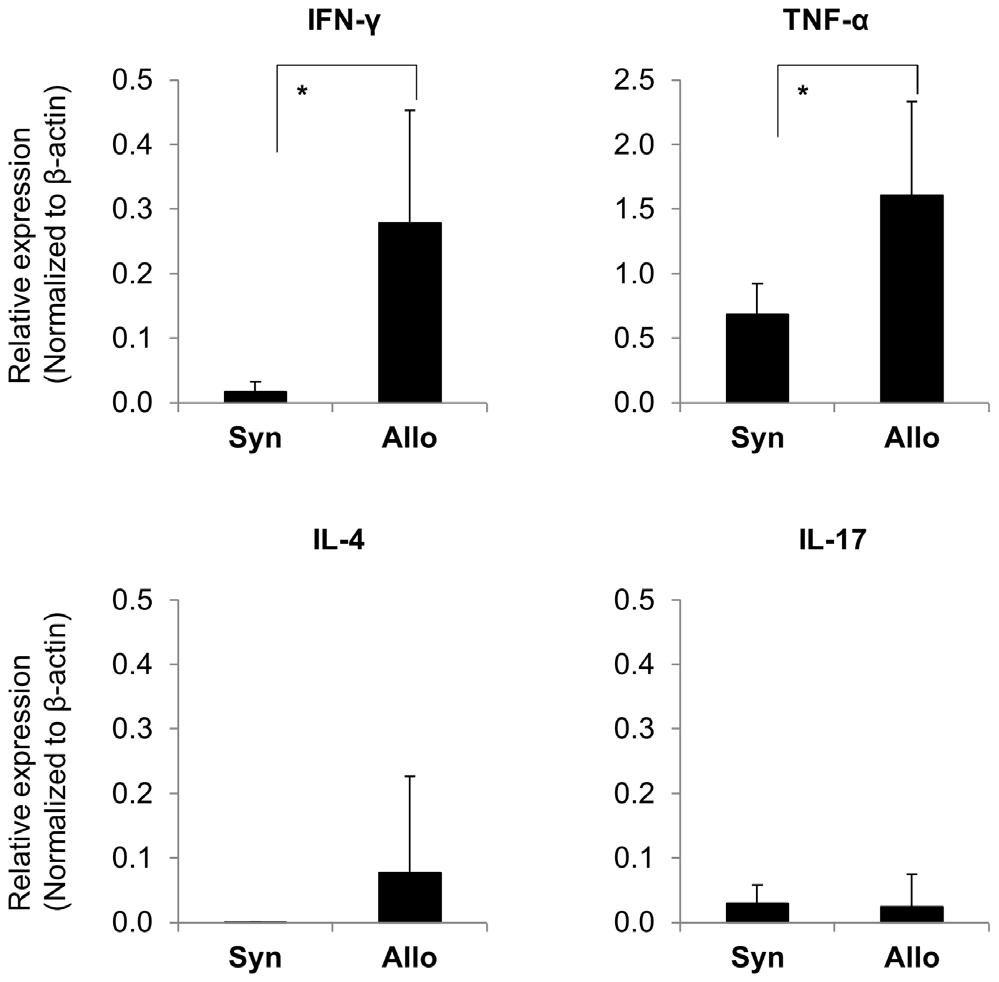
Real-time reverse transcription-PCR analysis of cytokines in the kidney after bone marrow transplantation (BMT). The expression of interferon-γ (IFN-γ) and tumor necrosis factor-α (TNF-α) was significantly up-regulated in the kidney on day 28 in allogeneic (Allo) BMT rats compared with that in the syngeneic (Syn) BMT rats. The expressions of interleukin 4 (IL-4) and IL-17 were not significantly different between these 2 groups. *P<0.05.

On day 28, infiltrating cells in the kidney were characterized by a large number of CD3+ T-cells and ED1+ macrophages in the interstitium (Fig. 7). CD3+ T-cells were mainly constituted with CD8+ T-cells (Figs. 7, 8A–C). CD4+ T-cells also infiltrated the kidney (Fig. 8D–F). Almost all infiltrating mononuclear cells involved in renal inflammation originated from the donor transplant (Fig. 8G–I). Only a minimal esterase+ neutrophils were present in inflammation (data not shown). In syngeneic BMT and non-BMT control rats, minimal numbers of CD3+ T-cells, CD8+ T-cells, and ED1+ macrophages infiltrated the kidney. IgM, IgG, and C3 depositions were not seen in the kidney (data not shown). The expression of rat MHC class II significantly increased in renal tubules in allogeneic BMT rats than those in syngeneic BMT and non-BMT control rats on day 28 (Fig. 7). In cytokine milieu in the kidney, the expressions of INF-γ and TNF-α were significantly increased on day 28 in allogeneic BMT rats compared with those in syngeneic BMT rats (Fig. 9). The expressions of IL-4 and IL-17 did not increase in the kidney in allogeneic and syngeneic BMT rats.

## Discussion

The occurrence of GVHD is a major complication after HCT. In the present study, although the skin, liver, and digestive tract were found to be the main target organs of GVHD, we demonstrated that the kidney could also be a direct target organ of acute GVHD following BMT. Pathological examination revealed infiltration of mononuclear cells, CD3+ T-cells, mainly CD8+ T-cells, CD4+ T-cells, and macrophages, in the renal interstitium with peritubular capillaritis, tubulitis, acute glomerulitis, and endarteritis. These findings suggest the development of T cell-mediated injury in the renal microvasculature and renal tubules during acute GVHD of the kidney. Increased urinary NAG level was an early marker of acute GVHD in the kidney, whereas serum Cr and urinary protein levels were stable. Acute GVHD in rat BMT model in the present study was different condition from the clinical human GVHD after HCT, because human GVHD is induced in rich stem cell transplantation with a very close degree of HLA matching between donor and recipient, and use the immunosuppressant or conditioning regimens to prevent GVHD. Therefore, further studies are needed to evaluate the acute GVHD of the kidney in human GVHD after clinical HCT.

After allogeneic HCT, GVHD is a major cause of morbidity and mortality, responsible for 15–40% of mortality [15], [16]. In acute GVHD, the most commonly affected organs include the skin, liver, and gastrointestinal system [17], [18]. In the present study, recipient Lewis rats also showed typical acute GVHD in the skin, liver, and intestine after allogeneic DA rat BMT. In contrast, in the Lewis-to-Lewis syngeneic BMT control rats, acute GVHD did not develop, even in the skin, liver, or digestive tract. In the kidney, acute GVHD is a common etiology and independently increases the risk for acute kidney injury [19]. However, GVHD can contribute to renal dysfunction indirectly through nephrotoxicity induced by a calcineurin inhibitor used in prophylaxis against GVHD, severe GVHD with diarrhea and dehydration, and cytomegalovirus reactivation [3], [5]–[7], [20]. However, it is generally accepted that acute GVHD of the kidney does not occur and acute GVHD itself cannot be involved directly in renal injury and dysfunction.

On the other hand, acute renal disorders after HCT are considered to be mediated by multiple factors [3], [5]–[7]. As patients with hematologic malignancy receive higher doses of chemotherapy and experience frequent infections during neutropenia, renal dysfunction may be attributable to GVHD as well as infiltration of underlying diseases [5]. High-dose chemotherapy and total body irradiation administered in the induction regimen may directly cause renal damage [21]. Rapid cytolysis of tumor and normal marrow can cause tumor lysis syndrome with renal injury due to hyperphosphatemia, as well as urate and xanthine nephropathy [22]. Infusion of cryopreserved marrow or blood progenitor cells may lead to renal insufficiency [23]. Post-transplantation infections often lead to acute renal insufficiency because they may be accompanied by hypotension and renal hypoperfusion. Antimicrobials used for prophylaxis and treatment of infections are also commonly nephrotoxic [3], [5]–[7], [22], [23]. In the present study, however, the relationship of all these factors with renal injury and inflammation could not be assessed, as our experiment did not use these nephrotoxic agents, except for lethal 10 Gy irradiation. In addition, the lethal 10 Gy irradiation could not have contributed to renal injury and inflammation in the present study, because syngeneic BMT rats that received lethal 10 Gy irradiation and syngeneic BMT showed minimal renal dysfunction and no obvious renal inflammation. Therefore, we considered that multiple factors excluding acute GVHD could not be associated with renal dysfunction and renal inflammation in our model.

Recently, several studies have reported that GVHD can involve renal insufficiency [8]–[11]. Membranous nephropathy after HCT may be associated with chronic GVHD [8], [9]. In a BMT mouse model of acute GVHD, in vivo imaging of the mice revealed that several non-classical organs are infiltrated by cytotoxic T-cells during GVHD, including the brain, kidney, and connective tissues [11]. In autopsy cases after HCT, allogeneic HCT recipients with severe GVHD tended to have tubulitis and peritubular capillaritis [10]. These studies may suggest that some renal dysfunction is associated with GVHD.

In the present study, we found significant infiltration of donor leukocytes in the kidney, and that infiltration of CD3+ T-cells, CD8+ T-cells, CD4+ T-cells, and macrophages mediated renal inflammation with peritubular capillaritis, tubulitis, acute glomerulitis, and endarteritis in allogeneic BMT recipients with systemic acute GVHD. Our findings of acute GVHD in the kidney were quite similar to pathological findings, as acute T cell-mediated rejection of the kidney in allogeneic renal transplantation [24]. In allogeneic renal transplant rejection, the pathology of tubulitis and peritubular capillaritis, acute glomerulitis, or endarteritis is considered the T cell-mediated immune injury for renal tubular epithelial cells and renal microvascular endothelial cells, respectively [25], [26]. The expression of MHC class II in renal tubules significantly increased in acute renal GVHD in the present study, and it showed similar findings to acute T- cell-mediated rejection in the kidney transplantation. Therefore, we considered that the pathology of the kidney in acute GVHD in the present study indicated T cell-mediated immunologic injury of renal tubules and renal microvasculature.

GVHD is caused by host-reactive T-cells derived from the donor bone marrow itself, or from the peripheral blood that contaminates the BM during its preparation [27], [28]. Donor-derived CD8+ cytotoxic T-cells have been identified as key players mediating GVHD pathogenesis [29]–[33]. CD8+ cytotoxic T-cell levels in peripheral blood predict the development of acute and severe GVHD [34]. In addition, CD4+ helper T-cells are also important effector cells of GVHD [35]. In the present study, renal inflammation in acute GVHD was accompanied by infiltration of CD8+ T-cells and CD4+ T-cells. CD8+ T-cells in the peripheral blood seemed to be increased during the development of acute GVHD, although they rapidly decreased after the full development of acute GVHD, in allogeneic BMT rats.

In the GVHD pathophysiology, both cellular factors (such as donor T-cells and macrophages) and soluble factors (cytokines) play a role in the development of acute GVHD. Based on the cytokine profile, the Th1 cytokines (IFN-γ, IL-2, and TNF-α) have been implicated in the pathophysiology of acute GVHD [29]–[33]. The Th1 cytokines participate in the initiating events that culminate in GVHD, as well as amplify the disease process once established. The transcript levels of IFN-γ in CD8+ T-cells are a sensitive marker to detect active GVHD [36]. A series of clinical studies have demonstrated the correlation between circulating TNF-α levels or TNF receptor-1 levels following HCT and GVHD [37], [38]. In addition, several clinical studies have targeted TNF-α as part of a treatment strategy for acute GVHD [39]. In the present study, the expressions of IFN-γ and TNF-α mRNA increased in the kidney of allogeneic BMT rats compared with those in syngeneic BMT control rats. In our model, donor-derived CD8+ T-cells, CD4+ T-cells, and macrophages within Th1 cytokine milieu induced acute GVHD of the kidney that have classically been considered the main immune mechanism mediating GVHD pathogenesis. By contrast, in the present study, IL-4, one of the Th2 cytokines, was not significantly different between allogeneic and syngeneic BMT rats, which may be associated with the absence of antibody-mediated immune injury. Levels of IL-17 produced by Th17 cells, involved in many immunologic processes including several autoimmune diseases, were also not significantly different between allogeneic and syngeneic BMT rats.

Based on laboratory findings, serum BUN and urinary NAG levels increased in acute renal GVHD in the present model. Inflammatory damage to the renal tubules from GVHD may be associated with an increase in the urinary NAG levels. We speculate that urinary NAG levels may be an early marker of renal GVHD that can be detected when serum Cr and urinary protein levels are stable. Further studies are needed to clarify the occurrence of acute renal GVHD after clinical HCT, the correlation between acute renal GVHD and urinary NAG levels in human GVHD, and useful pre-emptive therapy to improve transplant outcome after clinical HCT.

In summary, the kidney may be a target organ of GVHD, and the increased urinary NAG levels after BMT may indicate the development of acute GVHD of the kidney. As the number of HCTs increases every year, hematologists, nephrologists, oncologists, and pathologists should work together to identify patients with acute GVHD of the kidney to both prevent its development and initiate therapy early to improve outcomes after HCT.
